# Application of Coordination Compounds with Transition Metal Ions in the Chemical Industry—A Review

**DOI:** 10.3390/ijms21155443

**Published:** 2020-07-30

**Authors:** Jacek Malinowski, Dominika Zych, Dagmara Jacewicz, Barbara Gawdzik, Joanna Drzeżdżon

**Affiliations:** 1 Faculty of Chemistry, University of Gdańsk, Wita Stwosza 63, 80-308 Gdańsk, Poland; jacek.malinowski@phdstud.ug.edu.pl (J.M.); dominikazych97@wp.pl (D.Z.); dagmara.jacewicz@ug.edu.pl (D.J.); joanna.drzezdzon@ug.edu.pl (J.D.); 2 Institute of Chemistry, Jan Kochanowski University, Świętokrzyska 15 G, 25-406 Kielce, Poland

**Keywords:** coordination compounds, chemical industry, polymer materials, technological processes

## Abstract

This publication presents the new trends and opportunities for further development of coordination compounds used in the chemical industry. The review describes the influence of various physicochemical factors regarding the coordination relationship (for example, steric hindrance, electron density, complex geometry, ligand), which condition technological processes. Coordination compounds are catalysts in technological processes used during organic synthesis, for example: Oxidation reactions, hydroformylation process, hydrogenation reaction, hydrocyanation process. In this article, we pointed out the possibilities of using complex compounds in catalysis, and we noticed what further research should be undertaken for this purpose.

## 1. Introduction

The use of complex compounds in processes employed in the broadly understood chemical industry is a very important aspect of the work of scientists around the world. Processes carried out on an industrial scale and conducted by scientists are enjoying the growing recognition of global corporations dealing in the production of various polymeric materials used in many aspects of our lives [1,2,3,4].

In order to understand the important function of coordination compounds in catalytic reactions during industrial processes carried out on an industrial scale, it is necessary to learn the exact mechanisms of the reactions carried out. Delving into the mechanism of an exemplary process will allow us to conclude that the most important changes occur in the coordination sphere of the comprehensive relationship. This means that many factors related to geometry, steric hindrance, or the used central atom and attached ligands of the complex compound affect the catalytic reaction.

In recent years, the focus has been on improving the efficiency and selectivity of processes. It has been confirmed that the selection of appropriate ligands in the metal coordination sphere allows a catalyst with properties that have been planned to be obtained. We can therefore conclude that the catalytic properties of the complex compound used depend to the greatest extent on the structure of the catalyst [5,6].

A very important aspect of work on chemical technologies is the smallest possible pollution of the environment, i.e., the application of the principles of green chemistry. Conducting reactions in organic solvents is replaced by supercritical liquids or ionic liquids. Developing the implement of biotechnological and organocatalytic methods allows environmentally friendly chemical technologies to be obtained [7,8].

In this publication, we present the latest methods of using coordination compounds in catalytic reactions used in the chemical industry. We present the influence of coordination compounds on the course of reactions taking place in organic synthesis (for example: Hydroformylation process, hydrogenation reaction, oxidation processes, olefin polymerization processes). We also describe the application of green chemistry principles in a catalysis with the participation of complex compounds.

## 2. Oxidation Processes

### 2.1. Wacker Process

**Figure 1:**
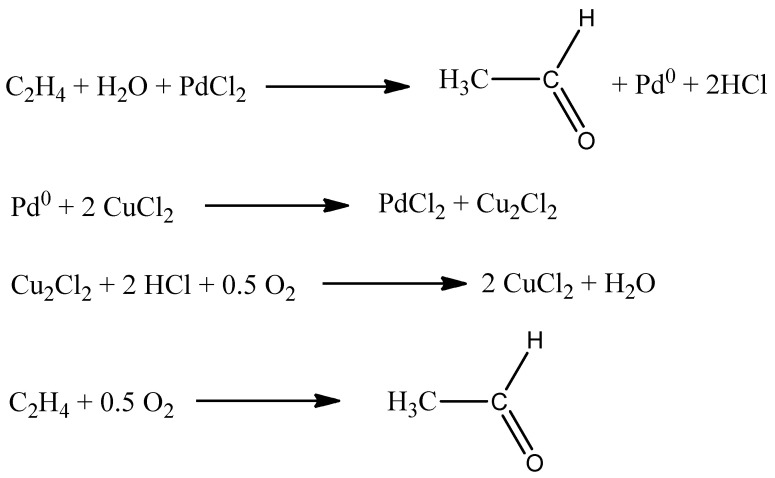
Reactions forming the catalytic cycle of the Wacker process [1].

The Wacker process was developed in cooperation of two concerns, Wacker and Hoechst, and then published in 1959. The process is a well-known reaction for obtaining acetaldehyde by oxidation of ethene. The reaction is carried out in an aqueous environment using a homogeneous catalyst (PdCl_2_ · CuCl_2_). The synthesis was recognized by the chemical industry, due to the fact that it was an alternative to the hydroformylation reaction. The acetaldehyde obtained in the synthesis is widely used. It is mainly applied for production of acetic acid, acetic anhydride or chloroform. In addition, it can form synthetic resins in condensation reactions with phenols and amines. The Wacker process is shown in Figure 1, in which stoichiometric reactions combine in one catalytic cycle. In the first stage, in an aqueous environment, it reacts with palladium(II) chloride, forming a stoichiometric amount of acetaldehyde, and palladium(II) is reduced to palladium(0). In the next stage, palladium(0) undergoes reoxidation in the presence of copper(II) compounds. Copper(I) is formed as the product of this reaction while in the last stage copper(I) is oxidized with oxygen to copper(II). Reaction 4 in Figure 1 represents the overall reaction of the process [9,10].

According to research recommendations, the oxidation stage is actually catalyzed by [PdCl_4_]^2−^, which is formed under the conditions of water and Cl^−^ ions. In the analyzed cycle, two chloride ligands are replaced by ethene and water. In the next stage, there is a nucleophilic attack of water on ethene in the complex, followed by substitution of the chloride ligand by another water molecule. β-elimination of a hydrogen atom leads to the formation of vinyl alcohol. This stage is followed by a series of processes leading to the production of acetaldehyde and palladium(II) hydride. Due to the fact that palladium(II) hydride is unstable, Pd(0) and HCl are formed as a result of the reduction reaction. The cycle is closed. Palladium(0) is oxidized to palladium(II) with CuCl_2_ [11,12].

In [13] the researchers carried out quantum mechanical tests and they concluded that the anti-nucleophilic attack is the step determining the Wacker process under standard conditions. Unfortunately, experimental studies did not confirm the results obtained by calculation methods. Keith et al. report that the Wacker process is determined by a syn-nucleophilic attack [14].

The Wacker oxidation usually gives ketones as reaction products, but the literature reports that one can carry out the Wacker process that will give 99% selectivity in obtaining aldehydes, e.g., using 1,4-benzoquinone, t-BuOH, and PdCl_2_ (MeCN)_2_ [15,16,17,18]. Regarding the selectivity of the Wacker process, products with high selectivity are also obtained using styrene derivatives as substrates with the reaction carried out under mild conditions [19].

Studies conducted over the years proved that the Wacker process produces environmentally hazardous, chlorinated by-products, as well as harmful copper waste. According to the principles of green chemistry, solutions are being sought that affect the protection of ecosystems and the whole world. Researchers at the University of Pune proposed the Pd(0)/C system as a heterogeneous catalyst that can be recycled. This allowed the elimination of pure CuCl_2_. In addition, they used potassium bromate KBrO_3_ as an oxidizing agent instead of molecular oxygen. By that, they developed a method using inexpensive substrates and achieved high process efficiency [20].

One may conclude that the by-products formed during the Wacker process are a major disadvantage of this cycle. Chlorine derivatives are environmental pollutants. Therefore, scientists should attempt to deactivate the resulting by-products.

### 2.2. Hydroperoxide Epoxidation Reaction of Olefins Catalyzed by Mo(VI) Complex

Epoxides are olefin oxides. Their structure contains a three-membered ring made of two carbon and oxygen atoms. They are the heterocyclic compounds. These compounds are characterized by high reactivity. For example, they undergo reactions that are accompanied by ring opening and closing followed by attachment of the nucleophile [21,22]. These reactions are used in the production of hypertension medications, antibiotics, and steroids. Hydrogen peroxides are the most common oxidants in the olefin epoxidation processes; H_2_O_2_ is rarely availed because it poorly dissolves in hydrocarbons. The course of the process is catalyzed by metal oxides of groups 4–6, e.g., Ti, V, Mo, W. There are many methods of epoxide synthesis, but the most well-known is the propene epoxidation reaction. According to data, propene oxide production in the world is close to 6 million tons per year [12].

One of the epoxidation methods, which includes peroxide metal compounds, is the reaction of olefin epoxidation with hydroperoxide catalyzed by the Mo(VI) complex with a peroxide ligand. Olefin is attached to the active form of the catalyst, namely the ROO group. Olefin binds to the catalyst through an oxygen atom, which is a characteristic feature of this reaction mechanism. No bond is formed by the metal atom; however, molybdenum in the 6th degree of oxidation will have a positive effect on the breaking of the O–O bond and restoration of the catalyst [11,12].

The epoxy—propene oxide—formed in the reaction is commonly used for the production of polyether and polyhydric alcohols, ethers, or propylene glycols.

Studies on epoxidation using molybdenum(VI) compounds have contributed to the development of significant stereoselectivity and efficiency of conducted reactions. Unfortunately, the use of solvent-free epoxidation reactions remains to be explored.

### 2.3. Sharpless System for Asymmetric Epoxidation of Olefins with Hydroperoxide with Dimeric Titanium Complex as a Catalytically Active Form

**Figure 2:**
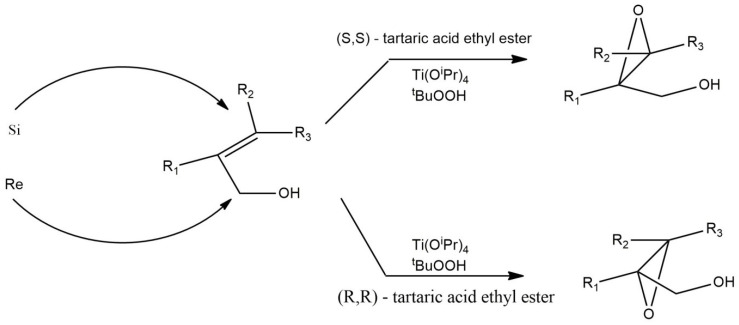
Sharpless system diagram for asymmetric olefin epoxidation [21].

The development of the chemistry of organometallic compounds contributed to the growing interest in the utilize of newly created systems. Sharpless conducted research on the use of organometallic catalysts in the asymmetric epoxidation reaction [23]. Allyl alcohol epoxidation is carried out with the participation of a system consisting of titanium(IV) isopropoxide, tartaric acid derivatives, and an oxidant in the form of tert-butyl hydroperoxide (Figure 2). Ti(O-^i^Pr)_4_ acting as a catalyst is a dimeric complex of titanium with tartaric ligands [21,24]. The tartaric acid ethyl ester used affects the stereochemistry of the reaction product. The ester interacting with the metal will form a complex. The resulting system undergoes the oxidation of hydrogen peroxide and the bond breaks on the appropriate side of the hydrogen bond depending on the chirality of the tartrate derivative [25,26].

The problem in the Sharpless system for asymmetric epoxidation of olefins with hydroperoxide with the dimeric titanium complex as a catalytically active form is the use of chlorinated solvents. For this reason, this reaction creates many problems in industrial production.

### 2.4. Cyclohexyl Hydroperoxide Decomposition Reaction Catalyzed by Cobalt(II) and Cobalt(III) Complex Compounds

Improving the technology of obtaining polymer fibers is the subject of research in numerous research centers. Caprolactam, which is used for the production of polyamide fibers and plastics, is particularly important in great chemical synthesis. According to statistical data, caprolactam production capacity is 5.2 million tons per year. It is worth noting that the cyclohexanol–cyclohexanone mixture from hydrogenated benzene has a huge share in caprolactam production in Poland and worldwide. It is estimated that 90% of the world’s production of this raw material uses this technology [27,28,29]. In Poland, the leading production process for caprolactam is CYCLOPOL technology developed at the Industrial Chemistry Research Institute in cooperation with Zakłady Azotowe in Tarnów and Puławy. CYCLOPOL technology has also been implemented in other countries, i.e., Russia, India, Spain, Italy, and Slovakia. In the first stage of the process, the hydrogenation of benzene to cyclohexane occurs. The cyclohexane oxidation process takes place in a bubble reactor. It is carried out in the liquid phase at a temperature in the range 155–165 °C, under pressure from 0.8 to 1.05 MPa. The technology implies that air or air enriched with oxygen should be an oxidant. A mixture of cobalt and chromium catalysts that support cyclohexane oxidation to a mixture of cyclohexanol and cyclohexanone and decomposition of hydroperoxides is also used. The next stage is the distillation of a mixture of cyclohexanols and cyclohexanone, followed by their dehydrogenation [12]. The key step is the cyclohexane oxidation reaction. The various cobalt compounds, e.g., naphthenate, play the catalyst role in this step. An important reaction step is the decomposition of cyclohexyl hydroperoxide. The reaction takes place according to radical mechanism [4].

Cobalt(II) and cobalt(III) compounds are described in the literature as catalysts in this reaction. The mechanism of catalysis is described by the Haber–Weiss reaction [4].

Co^2+^ + ROOH → Co^3+^ + RO^°^ + OH^−^

Co^3+^ + ROOH → Co^2+^ + ROO^°^ + H^+^

Ongoing work on improving the cyclohexane oxidation reaction node at the Tarnów plant has resulted in significant technological changes. The new CYCLOPOL–bis process consists of two stages. The process of synthesis of cyclohexyl hydroperoxides was separated from the process of their selective decomposition. In addition, optimal aeration, pressure, and temperature parameters were selected for these separate oxidation phases. The modernized CYCLOPOL–bis technology has allowed production costs to be minimized, the quality of products to be improved, and the effects on the environment to become less toxic [12].

Research to improve the selectivity of decomposition reactions is still needed. It should be emphasized that the CYCLOPOL–bis technology is close to the principle of green chemistry.

## 3. The Hydrocyanation Reaction

Hydrocyanation is used in the industry in the production of adipic acid nitrile. It is the basic raw material for the production of nylon 6,6, the reaction of which consists of attaching HCN to the olefin. DuPont company processes possess an economic significance, where adipic acid nitrile is obtained in the process of butadiene hydrocyanation. The process uses the Ni(0) catalyst with phosphate ligands [20].

The process consists of two stages. The catalyst plays a key role in the whole process. At first, the oxidative attachment of HCN to the NiL_4_ catalyst occurs and the hydrocyanide complex is formed. At this stage, the selection of a suitable ligand (L), e.g., a phosphorus ligand, is particularly important. Electronic and steric properties are taken into account, which have an impact on the course of individual stages of the hydrocyanation reaction. On the one hand, more basic ligands favor the addition of HCN. On the other hand, less basic ligands, which are characterized by high steric hindrance, are more popular [12,30]. Representative of this group is o-methylphenyl phosphate P(O-o-MeC_6_H_4_)_3_, which facilitates the reductive elimination of the product (3-pentenenitrile). According to the research, steric effects in the case of phosphine and phosphite ligands have a significant impact on the stability of Ni(0) complexes [31,32]. IR analyses determining changes of carbonyl vibration frequency (νCO) in [Ni(CO)_3_L] (where L denotes a broad variety of phosphorus ligands) complexes have shown that the nature of the ligand π acceptor increases the stability of Ni(0) complexes [33]. The steric properties of the ligands are represented using the Tolman cone angle. For ligands with a small cone angle of approximately 109°, dissociation will not occur even if we use high dilution solutions [31,32]. An example of such a complex is [Ni[P(OEt)_3_]_4_]. 16-electron complexes, e.g., [Ni[P(O-o-tolyl)_3_]], whose Tolman cone angle was 141°, and alkene complexes [Ni(alkene)L_2_] were also examined. Subsequent studies have determined that the increase in metal–alkene bond strength is affected by the replacement of the H-alkene atom by a more electronegative CN. The electron effect is visible here, which results in higher stability of the acrylonitrile complex. Tolman also presented in his researches ^1^H and ^31^P NMR studies of the complex [HNi(CN)L_3_] (where L is various phosphorus ligands) [34,35]. The addition of hydrogen cyanide to nickel(0) complexes is represented by the following reactions, where protonation is preceded by the dissociation of the ligand. The scientist pointed out that the addition of excess HCN may result in the formation of an undesirable complex [Ni(CN)_2_L_2_] (where L is a phosphorus donor ligand), which deactivates the catalyst. For this reason, [Ni(CN)_2_L_2_] is not active in the process of hydrocyanation [36,37,38].

$${\mathrm{NiL}}_{4}+\mathrm{HCN}⇌{{\mathrm{HNiL}}_{4}}^{+}{\mathrm{CN}}^{-}$$

$${{\mathrm{HNiL}}_{4}}^{+}{\mathrm{CN}}^{-}⇌{\mathrm{HNiL}}_{3}\mathrm{CN}+L$$

**Figure 3:**
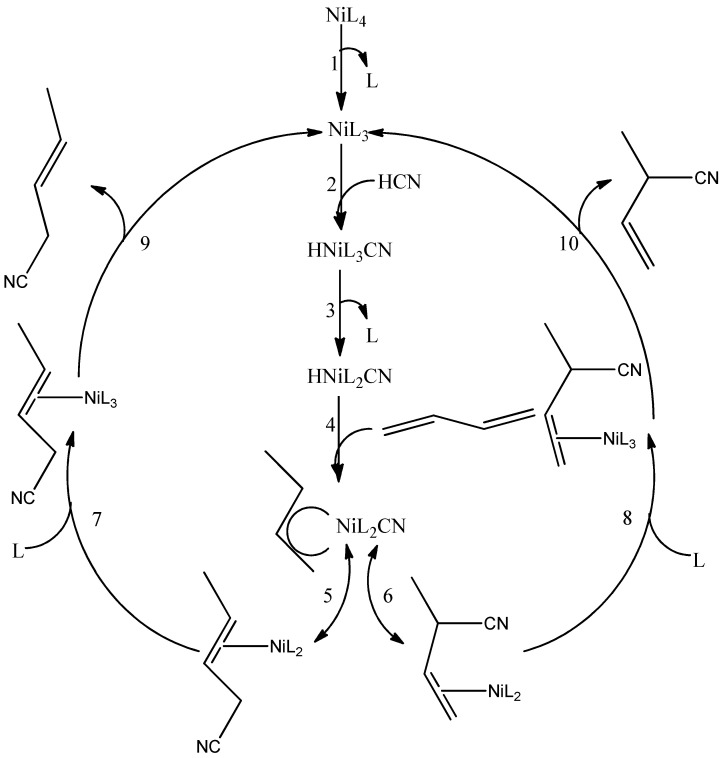
Mechanism of butadiene hydrocyanation [38].

Research concerns the HCN addition to butadiene at the first stage and the reaction taking place in the presence of a NiL_4_ catalyst (Figure 3). The reaction products are 3-pentenenitrile and 2-methyl-3-butenenitrile. The resulting isomer in branched form is not a substrate in the reaction of obtaining adipic acid nitrile; therefore, it is subjected to the process of isomerization to 3-pentenenitrile. The next stage takes place using the same catalyst with addition of Lewis acid. The Lewis acids used can be ZnCl_2_, ZnBr_2_, AlCl_3_, BPh_3_. It works with a free electron pair on the nitrogen of the CN group, which facilitates the creation of C–C connections. Isomerization of the double bond occurs and the 3-pentenenitrile is structured to an alkene (4-pentenitrile). The second CN group is joined in this reaction. Secondary by-products include 2-methylglutaronitrile (MGN), ethyl succinonitrile (ESN), and 2-pentenenitrile (AdN). Studies show that the hydrocyanation rate relative to isomerization is the highest for AlCl_3_ and decreases in the order of AlCl_3_ > ZnCl_2_ > BPh_3_ [12,29,30,38].

In 2019 it was confirmed that hydrocyanation can run without cyanide [39]. The mentioned reaction was asymmetric olefin hydrocyanation. In the alkenic hydrocyanation process, the use of bident phosphorus-based ligands is of great importance, which contributes to increasing the stability of the products obtained during hydrocyanation [40]. In addition to nickel complexes, ruthenium(I) complexes for hydrocyanation are used [41,42,43].

The important issue regarding the hydrocyanation reaction is alkene isomerization. Low reaction yields are a disadvantage of hydrocyanation reactions. These issues remain to be improved.

## 4. Metathesis Reaction

The first studies on the metathesis reaction date back to the 1930s. Schneider and Frolich described the pyrolytic conversion of propene to ethene and butene, which took place without the use of a catalyst at a high temperature of about 852 °C [44]. Metathesis has been a major course in academic and industrial research for years. Banks and Baile’s research, conducted in 1964, on the alkylation of isoalkenes with olefin on heterogeneous catalysts [Mo(CO)_6_/Al_2_O_3_, WO_3_/SiO_2_, Re_2_O_7_/Al_2_O_3_] at 160 °C turned out to be a breakthrough. These studies were aimed at obtaining fuels with a higher octane number, and also brought new information on the disproportion of linear olefins. Based on the developed technology, Philips Petroleum began production of high purity ethylene and but-2-ene from propylene [45]. Similar studies were conducted by Natta, who proved that the presence of homogeneous catalyst [TiCl_4_/AlEt_3_, MoCl_5_/AlEt_3_, WCl_6_/AlEt_3_] cyclobutenes and cyclopentenes allows for the polymerization of the ring opening metathesis polymerization (ROMP) type already at low temperature. His work concerned the copolymerization of ethylene with both linear and cyclic olefins and the polymerization of cyclic alkenes (ring opening metathesis polymerization; ROMP). Catalytic systems based on metal oxides on a solid support were also known. Due to the low cost of their production, and thus their wide availability, they were used in the Shell higher olefin process (SHOP) and in further exploitation of advanced Shell technology (FEAST) [46,47].

At the same time, numerous studies were being conducted, each of which was characterized by the use of compounds containing multiple bonds. In addition, the reactions mentioned took place under the influence of catalysts. The research aimed to find out the exact reaction mechanism that could explain the experimental results obtained. Researchers tried to describe the individual reactions with understanding of the structure of the transition state. They strived to characterize the structure of the catalytic center and determine which homo- or heterogeneous catalysts affect the conversion of compounds containing multiple bonds.

The creator of the term “metathesis” is Calderon, who used the synthesized heterogeneous catalytic system WCl_6_/EtAlCl_2_/EtOH to synthesize but-2-ene and hex-3-ene from pent-2-ene [48,49].

The exact definition of metathesis was provided by the Nobel Prize winners in chemistry: Grubbs, Schrock, and Chauvin (2005). Metathesis is a catalytic reaction accompanied by an exchange of double bonds between carbon atoms [12,50]. This reaction can be applied to various chemical compounds, for example: Internal, terminal, and cyclic olefins, as well as dienes and podiums. Metathesis reactions usually do not require high temperatures and pressure. In addition, all atoms are used in metathesis processes, and the resulting by-products can be processed or reused. Therefore, the reactions are reversible and meet the requirements of green chemistry [51].

The olefin metathesis process is considered to be an especially important technology for the production and processing of olefins. Considering the type of substrate used, we distinguish the following types of metathesis [51]:

- Cross Metathesis—CM
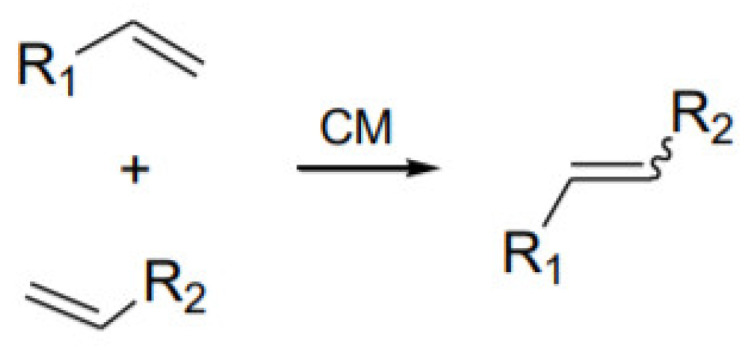

- Ring Closing Metathesis—RCM
- Ring Opening Metathesis—ROM

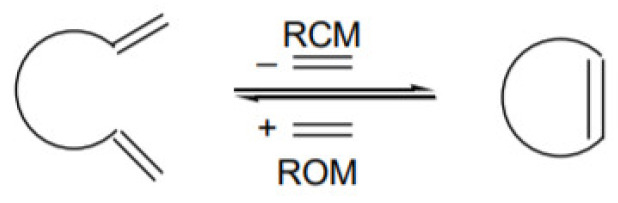

- Ring Opening Metathesis Polymerization—ROMP
- Acyclic Diene Metathesis—ADMET

**Figure 4:**
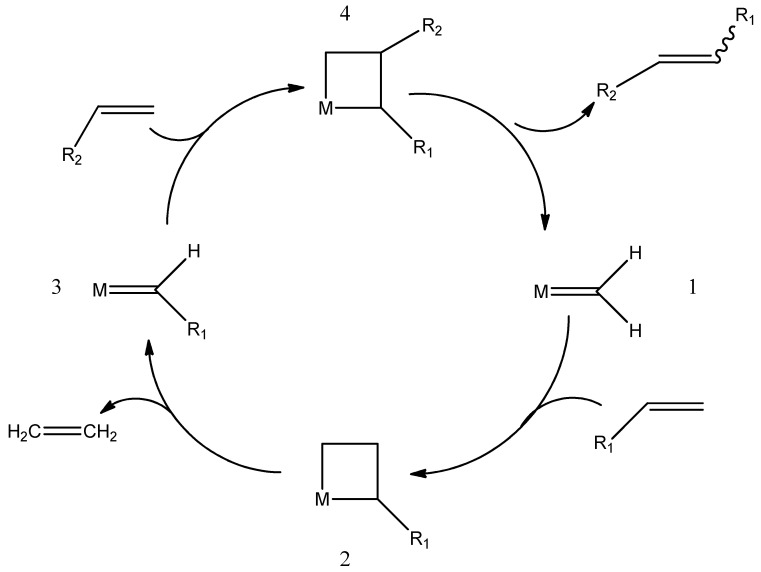
The mechanism of metathesis reaction.

Chauvin worked on the metathesis process with his student, Herisson. The mechanism of metathesis proposed by these colleagues is shown in Figure 4. They assumed that the metathesis reaction was catalyzed by alkylidene metal complexes. The first stage of the process is the production of metallycyclobutane **II** by reacting the R_1_-CH=CH_2_ substrate with the complex **I**. The resulting complex degrades to the alkene, in this case, ethene, and to the corresponding metallocarbene. The resulting metallocarbene **III** reacts with the olefin R_2_-CH=CH_2_, and the subsequent decomposition of **IV** leads to the formation of carbene **I**. Carbene as an alkylidene complex is a proper reaction catalyst [52,53].

**Figure 5:**
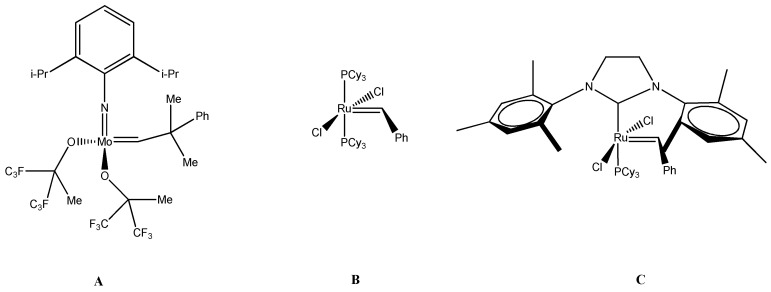
Metathesis catalysts, from the left: (**A**) Schrock’s molybdenum complex, (**B**) first-generation Grubbs catalyst, (**C**) second-generation catalyst.

**Figure 6:**
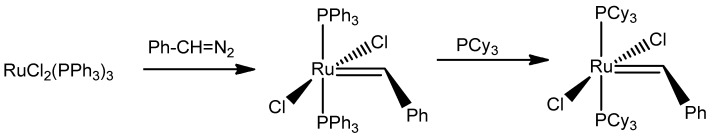
Grubbs catalyst preparation reaction.

Scientists in their research present homogeneous and heterogeneous catalytic systems. The metals used in the design of carbene complexes are transition metals with incompletely filled 3d, 4d, or 5d coatings [53]. Ruthenium, tungsten, molybdenum, and rhenium are most commonly applied. One of the first catalysts used in the described reaction was one-component tungsten carbide Ph_2_C=W(CO)_5_. The next step in the development of research on alkylidene catalysts was Schrock’s synthesis of a molybdenum complex (Figure 5). This complex showed high catalytic activity but was unstable. It was one of the first complexes that could be used without the activators, e.g., Lewis acids. It is worth noting that it is applicable for the synthesis of sterically crowded compounds. Disadvantages of this catalyst were sensitivity to oxygen, moisture, and polar groups. Complex compounds based on ruthenium are of particular interest. They show high reactivity towards olefins (C–C bonds) and tolerance to the functional groups of the substrates [53,54,55]. In addition, they are stable and do not react negatively to oxygen and moisture that may be present in the reaction system. An example of such a complex is the first-generation Grubbs catalyst (Figure 5). The catalyst synthesis is shown in Figure 5. The resulting complex, in addition to the above-mentioned features, is easy to store. It is one of the most commonly used complexes in metathesis reactions due to the fact that it can act toward obtaining carbo- and heterocyclic ring compounds, as well as macrocyclic compounds [54]. In addition, it catalyzes the metathesis cyclization reaction of enines and the alkene–alkyne metathesis. It is often used in polymerization reactions: ADMET, ROMP, and living polymerization of norbornenes [54]. It does not catalyze cross metathesis with α, β-unsaturated compounds, which is its drawback. Catalysts that contain imidazole derivatives are also noteworthy. N-heterocyclyl carbenes are obtained by reacting a first-generation Grubbs catalyst with a compound that is a source of stable carbene, e.g., the imidazolidine salt (Figure 6). In comparison to the first-generation catalyst, it takes part in reactions with α, β-unsaturated olefins, catalyzes ROMP cycloolefins with low ring stresses, and shows much higher activity.

Nickel complexes with chelate ligands have been used in metathesis reactions in a multistage SHOP process [54,55]. Initial material in this reaction is ethene. With the participation of a nickel catalyst, ethene is oligomerized and C_4_-C_40_ olefins are formed. Light < C_6_ and heavy > C_18_ olefins in the isomerization process are converted into appropriate internal olefins. In the next stage of the metathesis process, olefins of medium length C_11_–C_14_ are formed from them. The obtained internal olefins are used in the production of surfactants. In addition, the olefins subjected to a carbonylation process and hydrogenated to alcohols are the substrates in the synthesis of alkyl-benzenes. The separated C_6_–C_18_ fraction is used for the production of plasticizers, detergents, and also lubricants. The oligomerization catalysts are the nickel complexes mentioned above. Chelate ligands are coordinated by oxygen and phosphorus atoms. Scientists pay particular attention to the hemilability of metal-coordinated ligands. Such ligands have the ability to open and close the ring, which is possible due to the different strength of coordination of atoms that act as donors. This property allows transitions between cis- and trans-forms, and the change in symmetry affects the selectivity of the reaction. Due to the selectivity, scientists are conducting numerous studies on the synthesis of catalysts, affecting the preparation of sterically crowded olefins and catalysts, which control the stereoselectivity of reactions [55].

The following complexes are olefin metathesis catalysts: Heterocyclic carbene-coordinated compounds of ruthenium, salamo-type bisoxime complexes of Co (II) and Ni (II), carboxylate, phenolate, hydroxycarboxylate, and catecholate derivatives of Ti (IV) [56,57,58]. It should also be noted that FeCl_3_ also catalyzes the metathesis reaction [59,60].

In our view, the metathesis reaction provides many opportunities for the creation of various chemical compounds. It has application in refining industry and chemical studies. It is a powerful method of synthesis. For this reason, it is worth looking for new catalysts that meet the principles of green chemistry that can be used in metathesis.

## 5. Hydrosilylation Reaction

### 5.1. Reduction Pt(II) to Pt(0) in Reaction with Silane HSi(OEt)_3_

Silicon compounds are widely used in the modern world. They are applied as additives to cleaning products, cosmetics, and even food products. In addition, they increasingly have found application in electronics and the automotive industry. For example, organosilicon polymers, polycarbosilanes, and polycarbosiloxanes are used as precursors of ceramic materials and are resistant to chemical agents. Some of these materials exhibit optoelectronic, photoconductive, or electroluminescent properties. The synthesis of silicon derivative compounds, necessary for the production of the above materials, is based on the hydrosilylation reaction. Hydrosilylation is the catalytic addition reaction of organic and inorganic hydrogen silanes to unsaturated compounds. Depending on the substrate used in the synthesis, new Si-C, Si-heteroatom bonds are formed. The most commonly used hydrosilylation catalysts are platinum(II) complexes; however, other transition metal coordination compounds are also known, e.g., Pd(II), Co(II), Ni(II), Ir(I), Rh(II), and Ru(I). Scientist reports inform about numerous homogeneous catalysts active in the hydrosilylation reaction. They are [PtCl_2_(cod)], [Pt(CH_2_=CHSiMe_2_)_2_O_3_], [RhCl(PPh_3_)_3_], [Pd_2_(dba)_3_], where cod—cyclooctadiene, dba—dibenzoylacetone [61].

**Figure 7:**
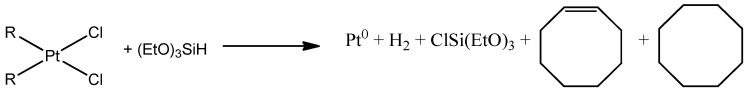
Example of the reduction of platinum(II) to platinum(0).

As an example of the reaction of reducing platinum(II) to platinum(0) is the reaction of [PtCl_2_(cod)] with (EtO)_3_SiH (Figure 7). The metal colloid formed in the reaction can also be used in the hydrosilylation process.

It should be noted that the hydroformylation reaction is commonly used in the industry for siloxane and silanes production. Chemoselectivity of hydroformylation means that its applicability for the synthesis of new compounds is high. Currently, hydroformylation catalysts are not based on platinum due to the reduction of catalyst costs. The use of new catalysts in hydroformylation opens up the possibility of designing new transition metal compounds with catalytic properties in this process.

### 5.2. Karstedt’s Catalyst

**Figure 8:**
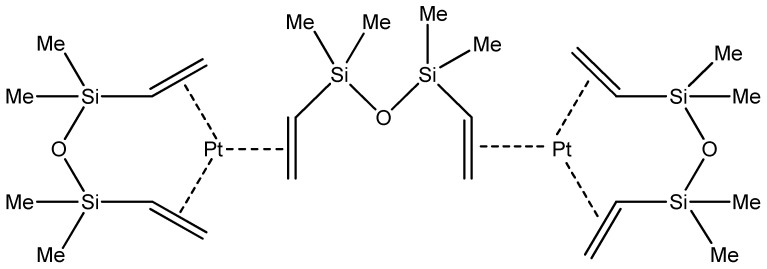
Karstedt’s catalyst.

Platinum complexes are most commonly used in hydrosilylation of carbon–carbon double bonds. Karstedt’s catalyst was described in 1973 as a compound of platinum(0) with divinylsilaxane ligands. It is formed in the reaction of divinyltetramethyldisiloxane with chloroplatinic acid, and it is applicable in numerous reactions due to its properties, i.e., high catalytic activity and good solubility in polysiloxane systems (Figure 8) [61,62].

Therefore, the high activity and selectivity of Karstedt’s catalyst causes it to be successfully used in industry. It will be difficult to find a replacement for this hydroformylation catalyst.

### 5.3. Hydrosilylation Mechanism Proposed by Chalk and Harrod and Modified Mechanism of Chal–Harrod—Catalyzed by Rhodium Complex Compounds

Reactions entering the cycle of the hydrosilylation process may take place with the participation of free radicals or in the presence of catalysts in the form of transition metal complex compounds. It is worth paying attention to the mechanisms of the hydrosilylation process of carbon–carbon multiple bonds in the presence of transition metal complexes. The use of transition metal complexes as catalysts affects the selectivity and speed of individual reactions. The hydrosilylation process is widely used in the synthesis of organosilicon compounds. In the 1960s, Chalk and Harrod introduced the first concept of a hydrosilylation mechanism [61]. In their research, they used hexachloroplatinic acid H_2_[PtCl_6_] as a homogeneous reaction catalyst. In the first stage, silane is attached to the catalyst metal. The configuration of the transition metal used in the catalyst is usually d^8^ or d^10^ [61]. The second stage leads to a stable alkene complex. Olefin coordination will only occur when the electron cloud transfer from the double bond to the hybridized metal orbit occurs. It is important to use excess substrate (olefin). As a result of the oxidative addition of HSiR_3_ to the complex, an M-H and M-Si bond is formed. The rearrangement of π–σ is considered a key stage. The migratory insertion of olefin for M–H binding generates a silyl–alkyl complex. The reaction product is formed by reducing the elimination of the alkyl complex [61,62,63].

**Figure 9:**
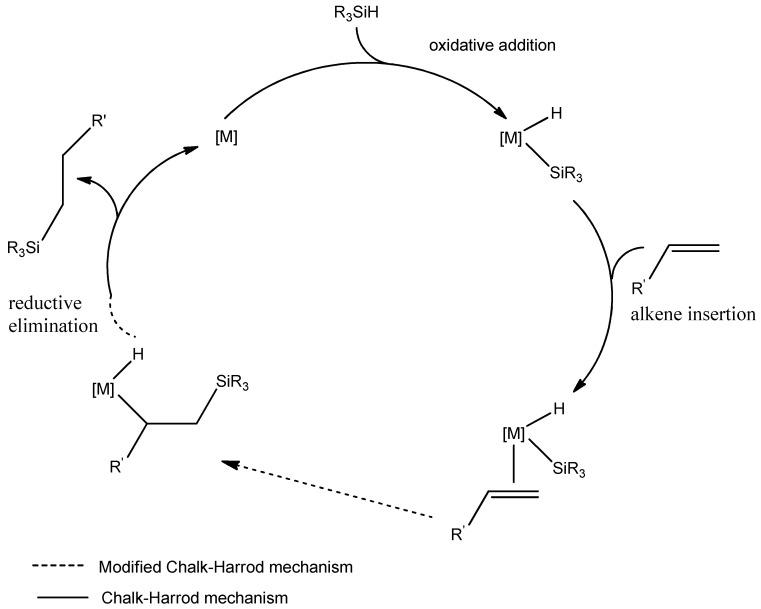
Hydrosilylation mechanism and modified mechanism proposed by Chalk and Harrod [63].

Research on the formation of unsaturated products in the hydrosilylation process was also undertaken. The explanation for this problem is the modified Chalk–Harrod mechanism, catalyzed by rhodium complex compounds. Although the product obtained in both processes is the same and does not differ in any way, the process itself changes. This is visible at the key stage which is alkene insertion. In the new process, it occurs to the M–Si bond, not to the M–H bond. The final stage proceeds in the same way by the elimination of alkenylsilane. Both processes are shown in the diagram (Figure 9) [61,62,63].

The hydrosilylation of alkenes with tertiary silanes can run without the use of a catalytic activator but with the use of the catalyst itself, i.e., cobalt(II) amide coordination compounds [64,65,66,67]. It is worth noting that alkene hydrosilylation can be successfully catalyzed by earth-abundant transition metal compounds [68].

The regioselectivity and stereoselectivity of the hydroformylation reaction depends on many factors e.g., solvent, catalyst, or temperature. Thus, it should be noted that the characteristic of the actual structure obtained product is difficult.

## 6. Hydroformylation Reaction

Hydroformylation is the reaction of olefins with hydrogen and carbon monoxide(II), where the products are straight chain (n) and branched chain (iso) aldehydes. This process occurs under the influence of the catalyst present during the reaction [69,70]. The products obtained in the form of aldehydes can be converted into alcohols, carboxylic acids, acetals, diols, or aldols under appropriate conditions. Most often, however, the intention of the reaction is to obtain an unbranched chain aldehyde. The catalytic system is most often characterized by the n/iso ratio. As we can see, the higher the numerical value of the ratio, the better the catalytic system used during the hydroformylation. The hydroformylation reaction scheme is shown below.

R-CH=CH_2_ + CO + H_2_ → R-CH_2_-CH_2_-CHO + R-CH(CH_3_)-CHO

The hydroformylation process was discovered by German chemist, Roelen, in 1938. He made the discovery during the Fischer–Tropsch reaction [71]. The first catalyst used during the hydroformylation reaction was hydridotetracarbonyl—[HCo(CO)_4_]. Then, the addition of phosphines to cobalt catalysts was applied, which resulted in a higher n/iso ratio and the hydroformylation process was carried out at a lower pressure and temperature.

A great breakthrough in the search for catalytic systems was made thanks to the work of the English Nobel Prize winner Sir Geoffrey Wilkinson. They allowed the discovery of compounds based on rhodium atoms that showed a thousand-fold higher catalytic activity in the hydroformylation reaction compared to cobalt catalysts [72,73].

Rhodium compounds, despite a higher price, are commonly used as catalysts for hydroformylation reactions. Union Carbide Davy Powergass Johnson–Matthey LPO—a British chemical company—uses a hydroformylation process using a precursor of formula [(acac)Rh(CO)_2_] and a modifying ligand, phosphine (PPh_3_). The addition of approximately 10% by weight PPh_3_ produces the active catalyst form—[(acac)Rh(CO)(PPh_3_)]. The hydroformylation process is carried out under the following conditions: At a temperature from 60 °C to 120 °C, pressure in the range of 10–50 bar. This process has a n/iso ratio of around 1:5. The hydroformylation process in Kędzierzyn–Koźle at Zakłady Azotowe is used in Poland [74,75].

**Figure 10:**
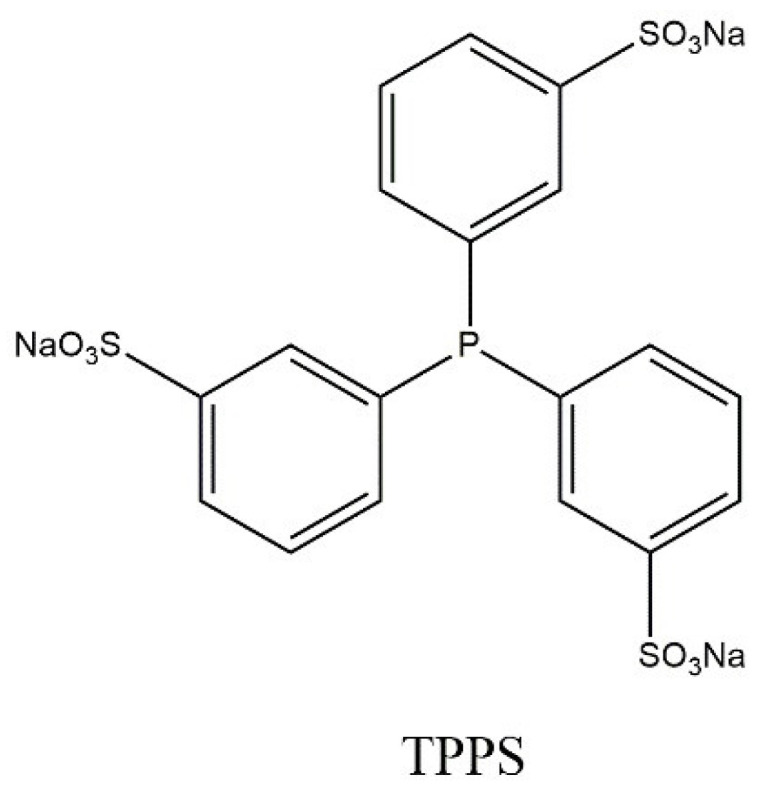
Simplified formula of sodium salt (tris(3-sulfonylphenyl)phosphine).

Kuntz’s discovery of the industrial conduct of the hydroformylation process and its commercialization by Ruhrchemie/Rhone-Poulenc is based on the use of a two-phase catalytic system based on a rhodium complex compound. Tris(3-sulfonylphenyl)phosphine sodium (TPPS) was used in the hydroformylation process (Figure 10).

The use of the aforementioned catalyst system allows easy separation of the catalyst from the desired products (aldehydes) because it is in the polar phase. Therefore, the catalyst is used in a continuous process. Conducting the hydroformylation process using a two-phase catalytic system allows for high selectivity and activity for the production of a linear product to be maintained [76].

The electron and steric properties of ligands attached to the rhodium atom influence the selectivity of the obtained product in the hydroformylation reaction. A larger amount of the linear product in the olefin hydroformylation reaction is obtained by using ligands with weaker basic properties—for example, pyrrolylphosphines or phosphites [77,78,79].

The steric properties of the ligands are evaluated based on the size of the conic angle, while for chelate ligands they are based on the grip angle, which determines the value of the angle between the P–M–P bonds present in the complex with the ligand. Studies on the impact of the grip angle on the selectivity of the reaction have shown that the equatorial–equatorial location of donor atoms in chelating ligands found in rhodium atom-based catalyst systems results in obtaining the best n/iso values. This means that the geometry of the respective rhodium complexes, in this case, for grip angles, is about 120°, exhibiting the geometry of the trigonal bipyramid.

The newest reports from scientists inform that the use of carbene and phosphorus ligand catalysts simultaneously attached to the rhodium atom in the coordination sphere results in obtaining a much better n/iso ratio. [RhH(NHC)(CO)P(OPh)_3_)_2_], where NHC denotes heterocyclic carbene, is an examples of such a catalyst [80,81,82,83].

The catalysis of the hydroformylation process is constantly evolving with regard to the use of rhodium compounds as catalysts [84]. Even a rhodium single-atom exhibits catalytic activity in hydroformylation similar to the RhCl(PPh_3_)_3_ complex [85]. Rhodium particles also have catalytic properties in hydroformylation [86,87]. In 2018 it was published that Fe(II) has the ability to catalyze hydroformylation of alkenes under mild conditions [88].

The most important advantage of the hydroformylation reaction is the ease of separation of the resulting product and catalyst. It is very important when receiving new materials.

## 7. Carbonylation Reaction

The carbonylation reaction is the process of attaching a carbon monoxide(II) molecule to an organic compound [89,90,91]. Most often, this process is used to produce acetic acid from methanol or an anhydrous derivative of the acid from the ester—methyl acetate. This reaction is carried out in the presence of transition metals, specifically metals in group 9, cobalt, rhodium, and iridium are used primarily. In addition, an iodide co-catalyst is needed during the process to activate methanol. This reaction produces acetyl iodide, which in subsequent hydrolysis produces a mixture of acetic acid and hydroiodic acid.

BASF carried out the first commercially conducted process of carbonylation of methyl alcohol with a cobalt catalyst. Then, industrial scale carbonylation of methanol was started by Monstanto, which applied a rhodium catalyst. The conditions operated by Monstanto compared to BASF differed in the use of lower carbon monoxide pressure and temperature. This process was also carried out with much better selectivity for methyl alcohol.

Studies on the mechanism of methanol carbonylation were conducted very thoroughly. Maitlis’ group examined the reaction using infrared spectroscopy and found that the mechanism of methanol carbonylation contains two catalytic cycles. The first cycle, rhodium, involves organometallic compounds and an iodide cycle containing organic reactions. The catalyst for this carbonylation reaction was the rhodium complex compound, cis-[Rh(CO)_2_I_2_]^−^ [92].

The methanol carbonylation process proposed by BP Chemicals, called the Cativa process, was carried out using an alternative iridium catalyst. The main difference of this process compared to the mechanism proposed by Monstanto is that it has about 100 times faster oxidation caused by the addition of CH_3_I to [Ir(CO)_2_I_2_]^−^ than to [Rh(CO)_2_I_2_]^−^.

However, a different type of catalyst is used when carbonylation of higher alcohols is carried out. This is caused by too slow a reaction using a rhodium catalyst. Therefore, palladium catalysts are applied that simultaneously also catalyze the olefin hydroxycarbonylation.

**Figure 11:**
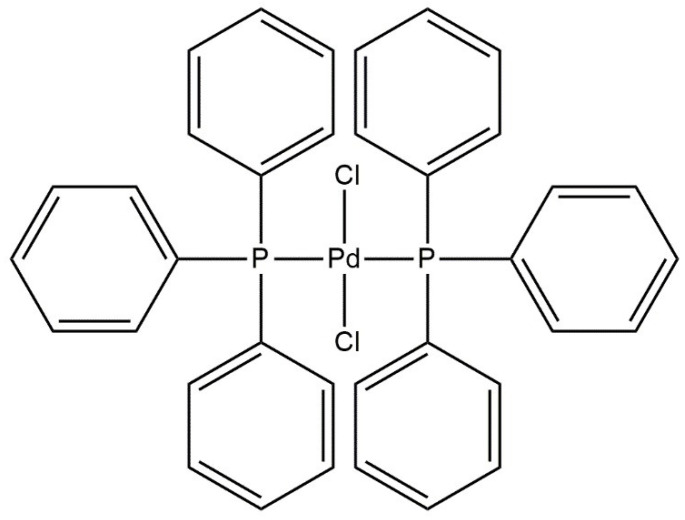
Simplified formula of the palladium complex used during ibuprofen synthesis.

The palladium complex compound-PdCl_2_(PPh_3_)_2_ is used in the carbonylation reaction, where the product is the known anti-inflammatory drug, ibuprofen. This synthesis is carried out by Boots–Hoechst–Celanese. Below, Figure 11 shows a simplified formula of this catalyst.

Naproxen is another very well-known remedy in which production palladium complexes are used as catalysts. This drug belongs to non-steroidal inflammatory drugs, which are obtained in a multistage synthesis. The palladium complex is a catalyst during stage 2—Heck reaction, and during stage 3—hydroxycarbonylation.

**Figure 12:**
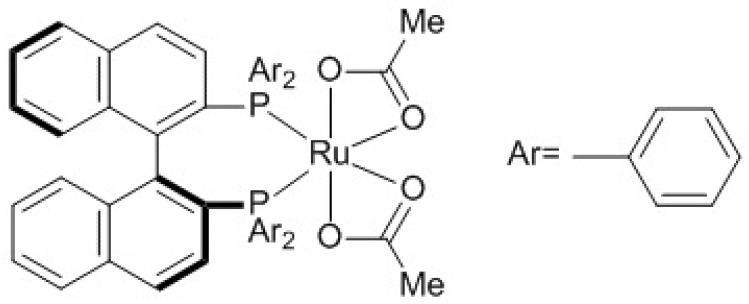
Ruthenium catalyst from S-BINAP used for the synthesis of S-ibuprofen.

Both ibuprofen and naproxen synthesis can be carried out in the presence of chiral phosphine. Reactions carried out in ionic liquids, using a ruthenium coordination compound as a catalyst, and with the introduction of a chiral phosphine allows us to obtain the appropriate optical isomer. Below, Figure 12 shows the catalyst used to obtain (S)-ibuprofen [93].

Hydroaminocarbonylation of olefins, aminocarbonylation of aryl iodides, and oxidative carbonylation of amines occurs with the application of bulk Pd catalyst without the use of organic ligands [94,95]. Iridium and immobilized-rhodium catalyze the methanol carbonylation process, leading to the formation acetic acid [96]. An interesting fact is that membranes, e.g., chitosan, polyamide, polyvinyl alcohol, are used to catalyze carbonylation reactions [97]. Membranes take part in the product separation of carbonylation. In 2019 K_2_[Fe(CO)_4_] as a catalyst promoting the formal insertion of CO was published [98].

Therefore, in the carbonylation reaction, iron(II) and platinum(II) complexes should be tested as catalysts. Palladium(II) complexes satisfactorily perform the role of catalyst carbonylation reaction; therefore, due to the similarity, analogous platinum(II) complexes should be tested.

## 8. Olefin Polymerization

Polymerization of olefins is a process that is used commercially around the world on a very large scale in the chemical industry. This is evidenced by the fact that the sum of the annual production of polyethylene and polypropylene is about 15 million tons. Another important aspect of using complex compounds such as olefin polymerization catalysts is the fact that it is economically cheap and environmentally friendly [99].

The newest version of the Ziegler–Natta catalysts used in the olefin polymerization process is the deposition of titanium(IV) tetrachloride on magnesium chloride. The MgCl_2_, the carrier, has a grain diameter of about 50 µm. A carrier with such grain diameter characteristics is obtained by precipitation from a soluble precursor solution with the addition of a Lewis base or by mechanical milling. The second stage is the addition of phthalic esters or silyl ethers. Magnesium(II) ions and titanium(III) ions have similar ionic rays, which make it possible to obtain a third-generation catalyst. This synthesis consists of adding titanium(I) tetrachloride to a solution of alkoxide, sulfide, or carboxylate containing magnesium(II) ions. Then the addition of TiCl_4_ and phthalates activates the catalyst [100].

Atom transfer radical polymerization (ATRP) is a technique that allows the production of polymers and co-polymers used commercially around the world. Polish chemist Matyjaszewski, working in the United States, discovered and developed this method of polymerization. The ATRP technique is cheap and does not pollute the environment in the production of polymeric materials on an industrial scale.

The application of radical transfer polymerization techniques allows for polymers with the appropriate structure and morphology to be obtained. Thanks to such control possibilities of the process being carried out, its application in various industrial technologies is becoming wider.

**Figure 13:**
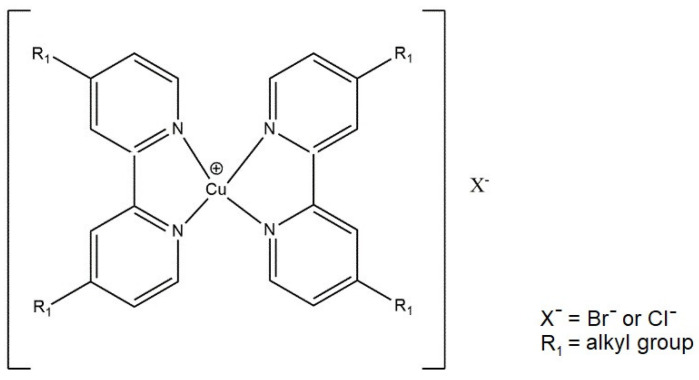
An example of a copper complex used as a catalyst in atom transfer radical polymerization.

Copper and iron coordination compounds are used as catalysts during radical atomization polymerization. Application of copper compounds during the process determines the necessity of the presence of reducing agents. The polymerization process carried out in water can be in homogeneous conditions or heterogeneous. Below, Figure 13 shows an example of a complex which is a catalyst for the ATPR process.

The technique of radical polymerization with atom transfer allows for the production of polymers that are components of moisturizing agents, surfactants, paints, and surfactants. It is also used in the synthesis of polar thermoplastic elastomers. It also allows the improvement of hydrophilic, antibacterial, or conductivity properties in the polymer materials produced.

**Figure 14:**
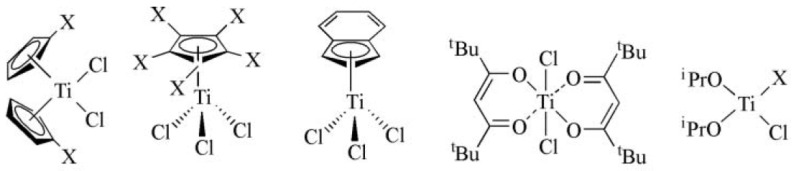
Titanium compounds used as catalysts in radical polymerization using organometallic compounds.

Radical polymerization using organometallic compounds (OMRP) is another technique used in polymerization. This method was initially proposed for the polymerization of acrylates. Acrylic acid, vinyl acetate, or other substrates are the monomers. Titanium, cobalt, iron, and chromium compounds have found application in this technique as catalysts. Figure 14 shows examples of ruthenium complexes used during OMRP [101,102].

In our view, olefin polymerization is a wide field for research, because nowadays there are reports of ever-increasing catalysts of this reaction being non-metallocene transition metal complexes [103,104,105,106,107,108]. The new complex compounds have higher values of catalytic activities than those traditionally used, and their synthesis is simple and cheap. New catalysts are chromium(III), oxovanadium(IV), and cobalt(II) complexes.

Late transition-metal catalysts are used in olefin polymerization. However, one should bear in mind that most of them cause the formation of amorphous and atactic polymers [109,110]. [Cu(2,3-pydc)(bpp)]·2.5H_2_O, [Zn(2,3-pydc)(bpp)]·2.5H_2_O, and [Cd(2,3-pydc)(bpp)(H_2_O)]·3H_2_O (2,3-pydc denotes pyridine-2,3-dicarboxyliate, bpp ***=*** 1,3-bis(4-pyridyl)propane) are metal–organic frameworks which are applied in heterogeneous catalysis [111]. Tridentate ligands based on N-, P-, and S-donors are used to synthesize the chromium(III) catalyst for ethylene trimerization and tetramerization [112,113].

One may conclude that this field must be developed because new catalysts can successfully replace traditionally used ones, which, unlike new catalysts, unfortunately did not meet the principles of green chemistry.

## 9. Hydrogenation Reaction

The hydrogenation reaction is a very important organic chemistry reaction. Hydrogen, one of the most important elements in the chemical industry, is used in almost 4% of hydrogenate carbon and hydrogen compounds. The mechanism of this reaction requires the presence of a catalyst, which is most often transition metal compounds. This process is carried out to create new bonds between carbon and hydrogen [114,115].

Heterogeneous catalysts are most often used during the hydrogenation reaction. The reason for their application is the ease of separation from organic products. However, the exploit of heterogeneous catalysts during the hydrogenation reaction is not possible in the asymmetric hydrogenation reaction. In addition, homogeneous catalysts, i.e., those made of complex compounds that are based on transition metals, increase chemoselectivity and regioselectivity of the reaction.

**Figure 15:**
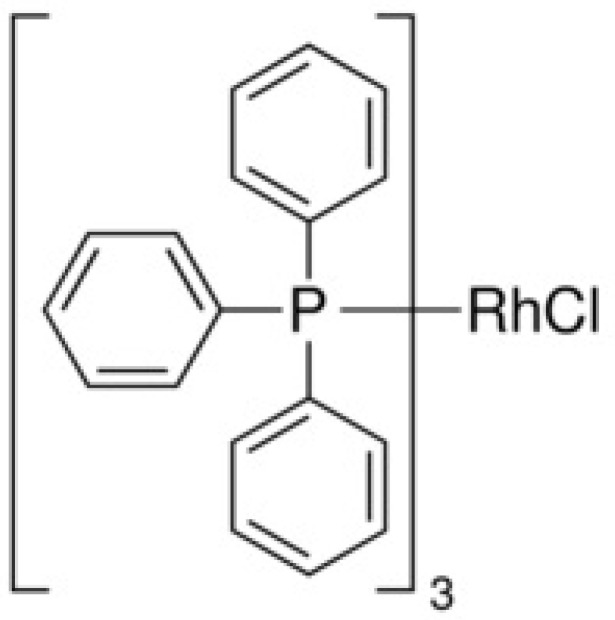
Simplified formula of Wilkinson’s catalyst.

If we use a metal complex as a catalyst during the hydrogenation reaction, this gives the opportunity to create a spatial and electronic structure of the compound. This can be achieved by choosing the right ligand. An example of the best-known hydrogenation reaction catalyst is the Wilkinson catalyst—[RhCl(PPh_3_)_3_] (Figure 15).

**Figure 16:**
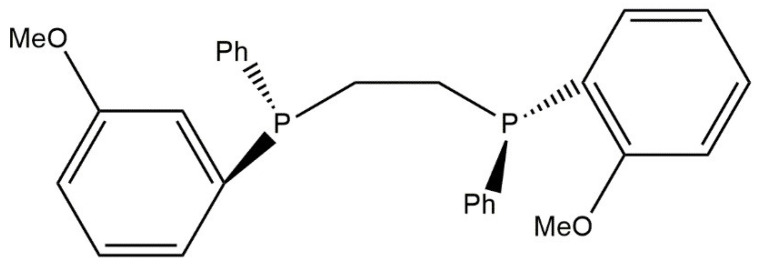
Simplified formula of the chiral form of phosphine-1,2-bis[(2-methoxyphenyl)phenylphosphine] ethane.

A very important enantioselective synthesis, during which the L-3,4-dihydroxylphenylalanine compound is obtained (L-DOPA), in one of the stages uses an asymmetric hydrogenation reaction. L-DOPA is a medicine applied during therapy against Parkinson’s disease. The need to obtain the appropriate product enantiomer informs us that the catalytic system of the hydrogenation mechanism must contain a chiral form of phosphine. During the synthesis reaction of L-3,4-dihydroxylphenylalanine, the catalytic system of the rhodium complex with 1,2-bis[(2-methoxyphenyl)phenylphosphine]ethane (DIPAMP) is used. Figure 16 shows a simplified formula of the chiral form of phosphine, which is a ligand in the rhodium complex [116].

It can be concluded that the use of phosphines as ligands in catalysts creates many opportunities to design new complexes as catalysts. Hydrogenation of esters and amides are catalyzed by these types of compounds. It is worth trying to synthesize new complexes containing other organophosphorus compounds as ligands, as well as trying to check the catalytic activity of phosphine-containing dual-core complexes.

## 10. Catalysis and Green Chemistry

### 10.1. Ionic Liquids—Catalytic Reactions

In modern catalysis, particular attention is paid to the principles of green chemistry. Technological processes must be as environmentally friendly as possible. Ethylammonium nitrate is a chemical compound recognized as one of the first ionic liquids. This chemical compound was obtained in 1914 by Walden as a result of the neutralization of ethylamine with the help of concentrated nitric acid(V) [117]. Ionic liquids are characterized by very low vapor pressure and low melting points. The low volatility of ionic liquids is a property that caused these chemicals to be classified as green solvents. The group of compounds called ionic liquids includes: Pyridinium, tetraalkylammonium, imidazolium, and phosphonium salts. Ionic liquids can be used as solvents friendly to the environment because it does not cause environmental load. The biodegradability and ecotoxicity of ionic liquids are currently studied. Ionic liquids are involved in the transport of metals and chemical compounds in soil [118].

Imidazolium salts can be used in two-phase systems. For this reason, they are the most commonly applied ionic liquids as a catalytic reaction medium. Ionic liquids are implemented in the technological process of obtaining S-Naproxen and S-Ibuprofen anti-inflammatory drugs. Asymmetric hydrogenation is catalyzed by ruthenium complexes with S-Binap chiral phosphine carried out in systems containing ionic liquid and alcohol, e.g., methanol.

Pyridinium ionic liquids are a very good medium for the methoxycarbonylation reaction of iodobenzene catalyzed by [PdCl_2_(cod)] or Pd(0)/PVP [119]. In the Sonogashira reaction, ionic liquids were used, and thus effective catalyst recycling was possible [PdCl_2_(P(OPh)_3_)_2_] [120]. Processes based on the Sonogashira reaction that applies in industries are the synthesis of alkyl derivatives. These types of reactions are used in the pharmaceutical industry. In this type of reaction tetraalkylphosphonium salts are the best reaction medium. The resulting product is separated from the ionic liquid containing the catalyst using hexane extraction. By-products are removed by washing with water.

Palladium precursors with triphenylphosphine in imidazolium ionic liquids are used in the Suzuki reaction to which bromobenzene undergoes [121]. In the Suzuki reaction, the carbene complex is the active form of the catalyst. The formation of carbene complexes is characteristic of ionic liquids with particular regard to imidazolium halides. Complex compounds of ruthenium(I) and palladium(II) undergo such reactions. During the reaction, the C(2) carbon of the imidazolium ring is deprotonated and an M–C bond is formed [122,123].

Carbene complexes are formed in situ if the ionic liquid is a solvent in the catalytic reaction. The reactivity of carbene complexes often differs from the catalyst precursor. For this reason, there are cases where the ionic liquid is an inhibitor of catalytic activity [123]. Iodobenzene methoxycarbonylation is an example of such a reaction. It can be concluded that ionic liquids are not only solvents, but are also active components of the reaction system. Ionic liquids take part in the formation of [IL]_2_[PdX_4_], where IL is the cation of the ionic liquid and X is the halide. [IL]_2_[PdX_4_] complex compounds are more active in the Suzuki reaction than in the [PdCl_2_(cod)] complex [124,125].

C–C coupling reactions catalyzed by palladium compounds in an ammonium salt environment, i.e., [Bu_4_N]Br, show that the use of ionic liquids increases the efficiency of the reaction [126].

The application of catalysts which are coordination compounds containing ionic liquids (as ligands) was implemented in the technological process of obtaining S-naproxen and S-ibuprofen. Therefore, we conclude that it is necessary to examine the influence of geometric isomerism of catalysts on their catalytic activity and the type of reaction product obtained.

### 10.2. Catalysts of Organic Reactions—Metal Nanoparticles

**Figure 17:**
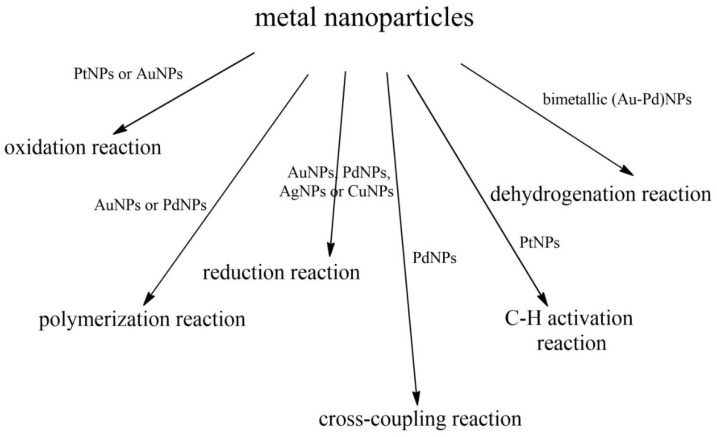
The application of exemplary metal nanoparticles in various catalytic reactions.

Metal nanoparticles have diameters in the range from 1 to 50 nm. The size of the nanotube has a very large impact on their properties. First information on the use of metal nanoparticles of Pd in Heck’s reaction comes from the 90s [127]. This concerned reactions between aryl bromides and styrene, or butyl acrylate and iodobenzene [127]. Metal nanoparticles obtained by reducing metal chloride salts in the presence of tetra-N-alkylammonium cations are electrostatically stabilized [127]. The application of nanoparticles and metal complexes in various catalytic reactions is presented in Figure 17.

Metal nanoparticles have some features of both homogeneous and heterogeneous catalysts. Nanoparticles can be a source of soluble metal complexes formed during a given reaction.

**Figure 18:**
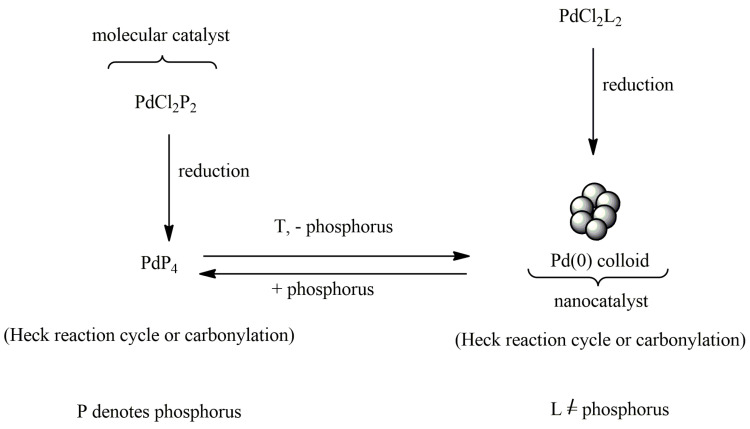
Transformations of palladium catalysts from monomolecular to nanoparticles. This figure is adapted from [129].

The complex compound undergoing a reduction reaction generates nanoparticles (Figure 18). Then the nanoparticles form a metallic structure because they agglomerate. The reverse process is also possible. This happens in the case of catalytic reactions during which nanoparticles are dissolved, which in turn contributes to the formation of complex compounds. There are known cases of equilibrium between nanoparticles and palladium complexes in the catalytic system. The equilibrium is particularly common when phosphorus ligands are present in the catalytic system [128]. Metal nanoparticles are formed as a result of a reduction of complex compounds. Reduction may occur during the catalytic reaction, but it is also possible before catalysis [129].

Among the nanoparticles, monometallic nanoparticles are the most popular. However, bimetallic nanoparticles are becoming the subject of more and more frequent scientific research. Cheaper base metals are part of the inner sphere of bimetallic nanoparticles [130]. The base metals are covered with a layer of precious metals, e.g., platinum, palladium, and ruthenium.

Catalysis with palladium and copper nanoparticles is applied to form C–C and C–S bonds [131]. The palladium nanoparticles cause coupling of vicinal–diiodoalkenes and acrylic esters and nitriles. The copper nanoparticles are used in chemoselective reduction of N-aromatic compounds.

It can be stated that the future research approach is the application of metal nanoparticles to identify catalytic sites [132]. The identification of catalytic sites for oxygen reduction reactions is extremely important when designing new highly active catalysts containing base metals.

## 11. Conclusions

This article describes many chemical technology processes that involve coordination compounds as catalysts. Many organic synthesis processes in the chemical industry, including the olefin polymerization process and atom transfer radical polymerization (ATRP) process, are used to produce polymer materials. The resulting products are used in many areas of our lives in the pharmaceutical industry—for the synthesis of drugs, polyethylene production, or for the synthesis of complex organic compounds. A very important aspect in chemical technologies is the widest possible application of the principles of green chemistry—in the case of catalytic reactions involving coordination compounds, biotechnological, or organocatalytic methods are applied. An example of the described principle can be use of ultrasound to better dissolve solutions or microwave radiation for heating.

The main conclusions of this review are following:

- PdCl_2_ taking part in the Wacker process forming a stoichiometric amount of acetaldehyde;
- Modernized CYCLOPOL-bis technology for caprolactam production allows for production costs to be minimized and the quality of products to be improved;
- Ni complexes with bidentate chelate ligands applied as catalysts in SHOP allow a separated C_6_–C_18_ fraction to be obtained, which is used for the production of plasticizers and detergents;
- [PtCl_2_(cod)], [Pt(CH_2_=CHSiMe_2_)_2_O_3_], [RhCl(PPh_3_)_3_], [Pd_2_(dba)_3_] are the hydrosilylation catalysts;
- Cu(I) and Fe(II) complexes with ligands such as halides, 2,2′-bipyridine, or tris(2-pyridylmethyl)amine are the catalysts in ATRP;
- [PdCl_2_(cod)] and Pd(0)/PVP are catalysts for the methoxycarbonylation reaction;
- K_2_[Fe(CO)_4_] catalyse carbonylation;
- [Cu(2,3-pydc)(bpp)]·2.5H_2_O, [Zn(2,3-pydc)(bpp)]·2.5H_2_O, and [Cd(2,3-pydc) (bpp)(H_2_O)]·3H_2_O are metal–organic frameworks which are applied in heterogeneous catalysis;
- [IL]_2_[PdX_4_] complex is more active than [PdCl_2_(cod)] in the Suzuki reaction.

In recent years, the development of research on the use of complex noble metal compounds as photoredox catalysts has been observed. This trend will continue for a long time because photoredox catalysis is a very useful method for activating small molecules.
